# The CEA/PCI ratio is a superior prognosticator than mCOREP for colorectal cancer patients with peritoneal carcinomatosis

**DOI:** 10.1016/j.sopen.2024.03.014

**Published:** 2024-03-28

**Authors:** Phelopatir Anthony, Shoma Barat, Nima Ahmadi, David Lawson Morris

**Affiliations:** a South Eastern Sydney Local Health District, Australia; b University of New South Wales, NSW, Australia

**Keywords:** Carcinoembryonic antigen, Peritoneal carcinomatosis, Colorectal cancer, Metastases, Prognosis

## Abstract

**Background:**

The CEA/PCI ratio, which evaluates tumour marker and burden, has been demonstrated as a prognosticator for patients with colorectal cancer with peritoneal carcinomatosis. The aim of this study was to compare the CEA/PCI ratio with the Modified Colorectal Peritoneal Score (mCOREP) for overall survival (OS) and recurrence free survival (RFS). There is no literature currently comparing both markers for RFS.

**Methods:**

Data was collected retrospectively for patients undergoing CRS and hyperthermic intraperitoneal chemotherapy (HIPEC) at the Peritonectomy Unit at St. George Hospital, NSW from January 2015 to December 2021.

**Results:**

From 187 patients, an increase in CEA/PCI ratio was associated with reduced OS (*p* < 0.01) and RFS (*p* < 0.01), whereas mCOREP score did not demonstrate such association with OS (*p* = 0.5) nor RFS (*p* = 0.4). However, CEA/PCI ratio greater than the median of 0.63 was correlated with an increased OS (*p* = 0.01), whereas the mCOREP greater than the median of 4 correlated with reduced OS (*p* < 0.01). Median mCOREP also demonstrated association with reduced RFS in patients with PCI <15 (*p* = 0.03), whereas CEA/PCI ratio above 0.63 demonstrated association with reduced RFS in patients with PCI ≥ 15 (*p* = 0.02).

**Conclusion:**

The CEA/PCI ratio is more associated with OS and RFS in patients with colorectal cancer with peritoneal carcinomatosis, when compared with mCOREP. CEA/PCI ratio above 0.63 was correlated with increased OS, whereas mCOREP above 4 is correlated with reduced OS. CEA/PCI ratio above 0.63 demonstrated reduced RFS for patients with higher PCIs. By contrast, mCOREP >4 illustrated reduced RFS in patients with lower PCIs.

## Introduction

The peritoneal cancer index (PCI) has long been established as an important prognostic tumour burden marker for determining which patients with metastatic colorectal cancer with peritoneal metastases are amenable for cytoreductive surgery (CRS) [1]. Indeed, patients with a PCI >15 have often been deemed as inappropriate for such management [2], with patients with a PCI between 12 and 15 having other parameters to be considered, such as chemosensitivity [3].

The CEA/PCI ratio has been suggested by Kozman et al. in 2017 [4] as a novel approach to calculate both tumour burden (PCI) and activity (pre-operative carcinoembryonic antigen) in a unified score, with potential prognostic impact for both overall survival (OS) and recurrence free survival (RFS). In addition, the modified colorectal peritoneal score (mCOREP) score has also been suggested as an independent prognostic marker for colorectal cancer patients with peritoneal carcinomatosis, with this score calculating tumour activity and systemic inflammation, with the utilisation of tumour markers, such as CA-199, CA-125 as well as C-reactive protein, platelet count, albumin and the presence of signet cell pathology being included here [5]. Initially, the mCOREP score was devised to determine predictability of open/close laparotomy.

This study aims to re-evaluate the prognostic impact of the CEA/PCI ratio, and compare it with the mCOREP score to determine which of these single index values demonstrates a stronger prognostic association with OS and RFS.

## Materials and methods

Colorectal cancer patients with peritoneal carcinomatosis who underwent CRS, with or without hyperthermic intraperitoneal chemotherapy (HIPEC) at St. George Hospital, Kogarah, NSW, Australia from January 1, 2015 to December 31, 2021 were included in the study cohort. Data was collected retrospectively through Electronic Medical Records (EMR), including age, sex, CA-199, CA-125. Indeed, all tumour markers, including pre-operative CEA, were collected within 2 weeks prior to operation. PCI was only determined from time of laparotomy. Presence of signet cell pathology, laboratory values and location of colorectal tumour were also collected. Endpoints of OS and RFS were also collected. Imaging was the only modality used to determine RFS, with RFS calculated from time of initial CRS till date of recurrence of peritoneal disease or on CT Abdomen/Pelvis or PET-CT.

All patients underwent pre-operative staging imaging, including and up to CT Abdomen/Pelvis, PET-CT and MRI-Liver with Primovist contrast. Each patient was also discussed in a multidisciplinary meeting with surgical oncologists, radiologists, medical oncologists and allied health staff.

The study was approved by the South Eastern Sydney Local Health District Ethics Committee.

### Inclusion criteria

Patients undergoing a maximum of one cytoreductive surgery during the study period were included in the cohort. Patients who underwent re-do CRS during the study period only had the initial CRS included, with all PCIs being included.

### Exclusion criteria

Patients who did not undergo a complete cytoreduction were excluded from this study.

### Statistical analysis

Data was analysed using IBM® SPSS® software Version 24 and Microsoft EXCEL®. Multivariate analysis was carried out using the SPSS® and univariate analysis for determining the significance of difference was calculated using GraphPad Prism. Incidence and Rate of incidence was reported for binary variables and reported as percent, standardised to the log of 10 [2]. Spearman's correlation was calculated to determine the correlation between variables.

The last follow-up date for cases was included in the survival calculations and marked as “lost to follow-up” from that time point, and censored from the overall participant cohort. Cox regression method for proportional hazard ratio was used to measure survival probability at given time (t) and calculated as part of the hazard function at time (t).

## Results

187 colorectal cancer patients with peritoneal carcinomatosis underwent CRS during the study period. The median age was 58.01 years. There was a female predominance with 111 females (59.36 %) and 76 males (40.64 %). 179 underwent HIPEC (95.72 %). Of the 8 patients who did not receive HIPEC, 4 had a PCI of 20 or greater, 2 demonstrated bladder involvement, 1 demonstrated root of mesentery involvement and 1 demonstrated a new pulmonary nodule. There were 96 overall deaths recorded (51.34 %). Recurrence was present in 112 patients (59.89 %). Median OS was 1177.5 days (38.64 months), whereas median RFS was 405 days (13.32 months). OS to 5 years numbered 92 patients, and 52 patients did not have recurrence within 5 years.

CEA was not available for 5 patients (2.67 %), CA 19–9 was not available for 6 patients (3.21 %) and CA-125 was available for all patients. The median CEA/PCI ratio was 0.63, with median pre-operative CEA and mCOREP each being 4. 81 (43.32 %) had a pre-operative CEA >5. Median CA-125 and CA-199 were 14 and 16 respectively, with a median PCI of 8 in this study. 31 patients (16.58 %) had a PCI greater than or equal to 15. 99 patients underwent systemic chemotherapy prior to cytoreductive surgery. The demographic data is displayed in Table 1.

**Table 1:** Demographic data relating to patient operative, clinical and pathological features.

	Number of patients (total n = 187) (%)	Median
Age (years)		58.01
Gender		
Male	76 (40.6 %)	
Female	111 (59.4 %)	
ASA		
I–II	35 (18.7 %)	
III–IV	145 (77.6 %)	
V–VI	0	
Missing	7 (3.7 %)	
Length of ICU stay		2
Length of hospital stay		16
Overall deaths	96 (51.3 %)	
Recurrence	112 (59.9 %)	
Recurrence free survival time (days)		13.3 months
OS at 5 years	92	
RFS at 5 years	52	
HIPEC	179 (95.7 %)	
CEA		4
Greater than 5	81	
PCI		8
Less than 15	156	
Greater than or equal to 15	31	
CEA/PCI ratio		0.6
mCOREP		4
Location of cancer		
Ascending colon	59 (31.6 %)	
Transverse colon	12 (6.4 %)	
Descending colon	14 (7.5 %)	
Sigmoid colon	47 (25.1 %)	
Rectum	12 (6.4 %)	
Synchronous	2 (1.1 %)	
Missing	41 (21.9 %)	
CA-125		14
CA-199		16
Pre-operative albumin		36
Pre-operative platelets		233
Signet ring histology	14 (7.5 %)	
Systemic Chemotherapy prior to CRS	99 (52.9%)	

### Associations of CEA/PCI ratio and mCOREP with OS and RFS

Amongst all available scores, an increase in CEA/PCI ratio was associated with reduced OS (*p* = 0.017), while mCOREP was not associated with OS (*p* = 0.623). As expected, increased PCI was correlated with reduced overall survival (*p* = 0.000). Amongst all scores, the CEA/PCI ratio was associated with reduced RFS (*p* = 0.017), in contrast with mCOREP (*p* = 0.414).

### Associations of median CEA/PCI ratio and median mCOREP with OS and RFS

CEA/PCI ratio above the median of 0.63 was associated with increased OS (*p* = 0.01), when compared with ratios below this score. However, this was not reflected with RFS (*p* = 0.34). mCOREP above the median of 4 showed association with both reduced OS (*p* = 0.00) and reduced RFS (*p* = 0.03). Additionally, mCOREP demonstrated correlation with an increased PCI (*p* = 0.002). The association of these prognostic markers with OS and RFS, both above and below their medians, are demonstrated in Table 2.

**Table 2:** Correlation of CEA/PCI ratio of 0.63 and mCOREP of 4 with both OS and RFS.

	Median time and confidence interval of OS (months)	Median time and confidence interval of RFS (months)
CEA/PCI ratio <0.63	31.9 (14.5–49.2)	20.3 (17.8–22.9)
CEA/PCI ratio >0.63	49.6 (32.2–66.9)	23.0 (20.4–25.5)
mCOREP <4	51.2 (32.4–70.0)	29.2 (19.3–39.0)
mCOREP >4	32.0 (13.3–50.8)	19.1 (9.2–29.0)

### Association of CEA/PCI ratio and mCOREP with RFS in patients with high (greater than or equal to 15) and low (<15) PCI

CEA/PCI ratio >0.63 showed association with reduced RFS in patients with PCI greater than or equal to 15 (*p* = 0.02), whereas an mCOREP >4 was correlated with reduced RFS in patients with PCIs <15 (*p* = 0.03), as displayed in Table 3, Table 4.

**Table 3:** Comparison of median CEA/PCI ratio and mCOREP, in both OS and RFS, with PCIs <15.

	Median time and confidence interval of OS (months)	Median time and confidence interval of RFS (months)
CEA/PCI ratio <0.63	38.6 (25.4–51.9)	22.6 (21.9–23.4)
CEA/PCI ratio >0.63	52.2 (38.9–65.4)	23.5 (22.7–24.2)
mCOREP <4	53.1 (37.6–68.6)	30.4 (19.6–41.1)
mCOREP >4	37.3 (21.8–52.8)	19.4 (8.7–30.2)

**Table 4:** (below): Comparison of median CEA/PCI ratio and mCOREP, in both OS and RFS, with PCIs >15.

	Median time and confidence interval of OS (months)	Median time and confidence interval of RFS (months)
CEA/PCI ratio <0.63	23.7 (17.4–30.0)	29.7 (13.1–46.3)
CEA/PCI ratio >0.63	17.2 (10.9–23.5)	12.7 (3.9–29.4)
mCOREP <4	24.8 (17.0–32.6)	14.3 (5.2–23.3)
mCOREP >4	16.8 (9.0–24.7)	23.5 (14.5–32.6)

### Association of CEA with OS and RFS

Increase in CEA was not associated with either reduced OS (*p* = 0.097) or RFS (*p* = 0.097), nor did it illustrate correlation with PCI (*p* = 0.358). Further, a CEA of 5 or greater did not reveal any association with either OS (*p* = 0.26) or RFS (*p* = 0.96).

## Discussion

While an elevated serum CEA (>5) has not demonstrated strong association with either OS or RFS in our study, multiple studies have indicated otherwise; Baqar et al. in 2019 [6] revealed that an elevated serum CEA of 5 was correlated with increased mortality and recurrence. However, the association between serum CEA and peritoneal carcinomatosis is not well established in the literature, as evidenced by Shimada et al. in a systemic review of serum tumour markers in patients with metastatic gastric adenocarcinoma [7]. In addition, our study did not demonstrate a linear correlation with CEA and PCI; indeed, the only tumour marker displaying association with PCI was CA-125. By contrast Baqar et al. [6] demonstrated that CEA, CA-125 and CA-199 were correlated with increased PCI.

Our study, however, did reaffirm the established association between increased PCI and reduction in OS and RFS.

Of note, our median CEA/PCI ratio of 0.63 is lower than our centre's 2017 median of 2.3, with both medians however yielding association with OS and RFS. This difference can be partially accounted for by the lower median CEA of 4 in this study compared with 7 by Kozman et al. [4], with the median PCIs being similar in both studies.

We included all patients with PCI of 15 and greater in the high PCI group, as patients with higher PCIs have been demonstrated to have shorter OS and RFS [8], and as such, including participants with these PCIs exclusively in this cohort may reveal new information for this patient group, in whom CRS may occasionally not be proceeded with [9]. Indeed, our study has demonstrated that the median CEA/PCI ratio is associated with RFS in patients with high PCIs, and as such could be espoused in the decision making to proceed with CRS for these patients. This finding serves as new evidence; Kozman et al. showcased that a CEA/PCI ratio of 2.3 held an increased prognostic impact in patients with low PCIs (10 or less).

As aforementioned, median mCOREP demonstrated association with both OS and RFS, with the median's prognostic impact for RFS more pronounced in patients with low PCI. These results are comparable to Enblad et al. in 2018 [5], who initially posited the modified COREP score. Indeed, their study elucidated that the mCOREP was associated with OS and risk of open/close laparotomy on univariate analysis only, with such findings not being reflected on multivariate analysis.

By contrast with our study, however, Enblad demonstrated no association between CEA/PCI ratio and OS (*p* = 0.1) and RFS (*p* = 0.3) on multivariate analysis, with CEA/PCI ratio only correlated with RFS on univariate analysis (*p* = 0.004). The discrepancies in our conclusions are to be noted in the context of our contrasting baseline demographic data. Indeed, Enblad's median PCI of 15 contrasts with our median PCI of 8, with a reduced median CEA/PCI ratio of 0.4, perhaps suggesting that Enblad's conclusion was made in a patient cohort with extensively more advanced peritoneal disease.

In their study, multivariate analysis for mCOREP only demonstrated correlation with RFS and survival <12 months. Of note, this study divided the cohort into 3 prognostic groups of unequal sizes: an mCOREP score of 0–5, 6–10 and ≥11. In this analysis, it was concluded that the median survival time for those with a score of 0–5 was 46 months, compared with 22 months for those with a score from 6 to 10. This contrasted from our study, however, our findings of a median OS of 51.17 months for an mCOREP score of 0–4 may align with the conclusion drawn by Enblad et al. In their study, however, patients were not grouped according to PCI. Given mCOREP has demonstrated with both OS and RFS in patients with low PCIs, these results may lend credence to the use of the mCOREP score in disease prognostication for these patients.

With this in mind, it is important to consider our study's limitations. Approximately 20 % of patients with colorectal cancer will not have an elevated CEA [10]. Additionally, being a retrospective cohort study, our study is prone to selection bias [11], with a limited sample size of 31 present for the high PCI group. It may be posited that ultimately, the mCOREP score is an exclusively pre-operative prognostic marker [12], whereas the CEA/PCI ratio has both pre-operative and intra-operative components; which may impede pre-operative selection. This may be addressed with the use of staging laparoscopy, however, this is a resource intensive exercise and not possible in select patients with significant adhesions [13]. Also, CT Abdomen/Pelvis is most proficient in detecting peritoneal carcinomatosis of 5 mm or greater [14], and as such may not adequately reflect true extent of recurrence.

## Conclusion

The CEA/PCI ratio is more associated with OS and RFS in patients with colorectal cancer with peritoneal carcinomatosis, when compared with mCOREP. CEA/PCI ratio above 0.63 was correlated with increased OS, whereas mCOREP above 4 is correlated with reduced OS. However, CEA/PCI ratio above 0.63 demonstrated reduced RFS for patients with higher PCIs. By contrast, mCOREP >4 illustrated reduced RFS in patients with lower PCIs.

## Ethics approval

Ethics approval was obtained from the South Eastern Sydney Local Health District Ethics Committee.

## Funding sources

This research did not receive any specific grant from funding agencies in the public, commercial, or not-for-profit sectors.

## CRediT authorship contribution statement

**Phelopatir Anthony:** Writing – review & editing, Writing – original draft, Investigation, Formal analysis, Conceptualization. **Shoma Barat:** Software, Methodology, Formal analysis, Data curation. **Nima Ahmadi:** Writing – review & editing, Supervision, Methodology, Investigation, Formal analysis, Conceptualization. **David Lawson Morris:** Writing – review & editing, Supervision.

## Declaration of competing interest

None declared.
